# Attitudes of Catholic Priests Regarding the Participation of People with Schizophrenia and Depression in Religious Practices: Relationships with Prejudices and Community Size

**DOI:** 10.1007/s10597-022-00953-7

**Published:** 2022-02-26

**Authors:** Lorenza Magliano, Gaetana Affuso

**Affiliations:** Department of Psychology, University of Campania “Luigi Vanvitelli”, Viale Ellittico 31, 81100 Caserta, Italy

**Keywords:** Attitudes, Prejudices, Schizophrenia, Depression, Priests

## Abstract

This study investigated whether priests’ attitudes regarding individuals with schizophrenia and depression participating in religious practices varied in relation to priests’ adherence to prejudices about these mental disorders (MD). A sample of 559 Italian priests completed a questionnaire on their views of either schizophrenia or depression. Data were analyzed using a multiple-group structural equation in which the diagnostic group was a moderator and the size of the municipalities in which the churches were located was a covariate. The study revealed that: priests’ attitudes towards churchgoers with MDs are related to views of these individuals as dangerous, easy to recognize and poorly aware of their MDs; community size has a direct effect on priests’ attitudes and an indirect effect through perceived dangerousness; the above-mentioned relationships do not differ by type of disorder. Sensitizing priests on stigma may be helpful to facilitate the participation of believers with MDs to religious practices.

## Introduction

Approximately 85% of people worldwide identify with a religion, mostly Christianity and Islam (Hackett, 2011). In line with epidemiological data, one out of four believers may suffer from a mental disorder (MD) in their lifetime (WHO, 2001). Several studies have shown a largely positive effect of religious practice on people with MDs (Bonelli & Koenig, 2013; Weber & Pargament, 2014). Religious faith and practice were found to be associated with milder psychiatric symptoms and better clinical and functional outcomes in MDs (Fallot, 2007; Mohr et al., 2012; Nolan et al., 2012; Russinova & Blanch, 2007; Shah et al., 2011; Webb et al., 2011). For people with MDs the religious community might represent a “second family” and a source of social interaction – acting as a facilitator in the recovery process (Griffith et al., 2016; Oman & Thoresen 2005; Smolak et al., 2013; Wong-McDonald, 2007; Yangarber-Hicks, 2004) - or a context fueling social isolation, loneliness, and stigma (Webb et al., 2011).

Within the religious community, priests go beyond a spiritual role, frequently acting as personal advisors in non-religious matters (Anshel & Smith, 2014). Moreover, priests promote religious and volunteer activities carried out by churchgoers. Priests have a more central role in the lives of small town or rural residents than urban residents, since religious congregations often serve as main social and community centers in less populated areas (Ellison et al., 2006). Because of the spiritual office they hold, priests are likely to be motivated by altruism and acceptance of all believers. However, as members of society, even priests may have stigmatizing attitudes towards people with MDs (Link et al., 1999; Pescosolido, 2013). Given the central role of priests in the acceptance of individuals with MDs within religious communities (Anshel & Smith, 2014), it is worthwhile to explore clergy attitudes toward people with these disorders and to develop strategies to improve those attitudes, if needed.

Studies investigating clergy attitudes toward the people with MDs found that priests’ attitudes differed according to their religion, the type of MD, and the priests’ levels of contact with individuals with these disorders. A survey on the attitudes of 32 male clergy from different religions (Leavey et al., 2007) reported that, despite an obvious sympathy with the plight of people with MDs, as lay people clergy had stereotypes and fears toward these individuals. Regardless of their frequent contact with churchgoers with MDs, only those clergy who had personal or familial experience with MDs felt comfortable with people with these disorders. Clergy who perceived violence as correlated with MDs, a perception more common in psychosis, were more reluctant to be engaged in the care of churchgoers with MDs. Exclusion of individuals with MDs from the church congregation ‘‘for the well-being of the community’’ was also reported. In a study of 25 Protestant seminary students (Stull et al., 2020), 32% viewed people with MDs as “childlike”, 40% thought that they were scary, 56% felt uncomfortable with them and 72% tended to avoid them. In a survey of 107 Christian and Muslim clergy (Igbinomwanhia et al., 2013), 71.1% claimed that people with MDs could be differentiated from “normal” people, and 68.2% stated that these individuals should be controlled like children. In a survey of 1031 US Methodist pastors (Lafuze et al., 2002), 53% perceived individuals with MDs as more dangerous than the average citizen. Moreover, clergy authoritarian attitudes towards individuals with MDs were negatively correlated with pastors’ contact with them. In a recent study by Aramouny et al. (2020) of 115 Christian clerics in Lebanon, 82.6% of participants believed that people with MDs needed to be controlled and disciplined as young children, 87.3% tended to avoid them, and 60.9% perceived them as dangerous. As commented by the study’s authors: “religious practice did not seem to protect against discrimination toward persons with MDs, suggesting that social representations of persons with MDs are so anchored that they resist the inherent empathy and humanistic nature of the clerics profession”. A recent survey carried out in Italy compared views regarding people with schizophrenia and depression in a sample of 559 Catholic priests (Magliano et al., 2021). The study results showed significant differences in priests’ opinions in relation to the type of disorder. Compared to depression group (N = 277), in the schizophrenia group (N = 282), priests were: more reluctant for churchgoers with this disorder to participate in parish activities and sacraments; more skeptical regarding the usefulness of prayer and the possibility of recovery; more convinced about the negative influence of the disorder on the affective life of the sufferers. Overall, the studies mentioned above have examined the views on MD of clergy in different religions and/or regarding different disorders. However, to our knowledge, no study has specifically investigated whether clergy views of people with MD influenced clergy attitudes regarding the participation of believers with MD to religious activities.

Using the dataset of the comparative survey described above (Magliano et al., 2021), in this study we investigated whether priests’ attitudes regarding the participation of individuals with schizophrenia and depression in religious practices varied in relation to priests’ adherence to common prejudices about people with these disorders. It should be noted that although the same dataset was used, there is no overlap in objectives, data analysis, or results between the two studies. The former examined differences in priests’ views between the two diagnostic groups; the latter focused on the relationships between priests’ beliefs about people with mental disorders and priests’ attitudes about the potential acceptance of people with these disorders in religious contexts. More specifically, we hypothesized that priests who were surer that people with MDs are: (i) dangerous; (ii) kept at distance by the others; (iii) unaware of their own condition; and, (iv) easy to recognize, would be: (v) more skeptical regarding the participation of churchgoers with MDs in parish activities and sacraments; and, (vi) more convinced that these individuals should be treated differently from other churchgoers during religious celebrations. To test the above-mentioned hypothesis, we used a multiple-group structural equation model. In the model, the diagnostic group - schizophrenia and depression - was considered as a potential moderator of the above-mentioned relationships, whereas the community size of the municipalities where the churches were located was included as a covariate potentially influencing all the other variables. Community size was included as covariate as it is used in population surveys to capture sociocultural differences between areas with different levels of urbanization in Italy (ISTAT, 2017).

## Methods

### Study Design and Participants

From February 2017 to December 2019, Catholic priests of parishes from six religious jurisdictions in Southern Italy were contacted personally or by phone by a researcher and invited to participate in a study on their views of MDs. Informed consent was sought in writing. However, several priests preferred to participate by giving verbal consent. Participants were asked to complete the Opinions on mental disorders Questionnaire, Priest version (OQ-P) (Magliano et al., 2021) after reading a randomly chosen description of either schizophrenia (13) or depression (14). Therefore, each participant completed the questionnaire only once and with reference to only one clinical description, either schizophrenia or depression. The questionnaire was self-administered either in the presence of the researcher at the parish church or in his/her absence, according to the participant’s preference. Information on priests’ socio-demographic variables, and experience with people with MDs were also collected. The study was approved by the Research Ethical Board of the Department of Psychology of the University of Campania “Luigi Vanvitelli” of Caserta, Italy (n. 22/2016 Department Board 6/12/16, and 16/2019 Department Board 14/5/2019) and conducted in accordance with the Helsinki Declaration.

Of the 609 contacted priests, 50 (8.2%) refused to participate in the study (reasons: lack of time and/or not interested – 66%; unwilling to give such information to people outside the religious organization – 12%; unknown – 10%; disagreement with research aims – 8%; no opinion regarding persons with MD – 4%) and 559 agreed to participate (91.8%). Of these, 282 completed the OQ-P after reading a description of schizophrenia and 277 completed the questionnaire after reading a description of depression. In both groups, most participants were middle-aged (53.04 ± 12.8 and 52.3 ± 13.9 years old), had a bachelor’s degree in theology (78.0% and 69.3%), and had been priests for over two decades (22.9 ± 13.8 and 22.4 ± 15.1 years). Nearly all participants (90.1% and 96.8%) stated they knew individuals with MDs attending the church to participate in celebrations (58.2% and 57.3%), religious groups (21.1% and 36.7%; *χ*^*2*^ 16.1, df 1, p < .0001) and voluntary activities (10.2% and 22.8%; *χ*^*2*^ 15.8, df 1, p < .0001), and to receive individual spiritual support (48.4% and 62.9%; *χ*^*2*^ 11.6, df 1, p<. 001), advices (45.8% and 59.6%; *χ*^*2*^ 10.2, df 1, p < .001), and economic help (27.3% and 34.8%). Further details on the descriptive data are reported in Magliano et al. (2021). In the two groups, the distribution of priests by community size of the municipalities where the parishes were located was as follows: 21.3% and 17.0% in ≤ 5.000 inhabitant municipalities; 5.0% and 6.9% in 5.001-10.000 inhabitant municipalities, 24.5% and 30.7% in 10.001-50.000 inhabitant municipalities, 19.5% and 16.6% by 50.000-100.000 inhabitant municipalities, and 29.8% and 28.9% by > 100.000 inhabitant municipalities. The classification by number of inhabitants is in line with those reported in population surveys by the Italian Institute of Statistics (ISTAT, 2017).

### Assessment Instrument

The Opinion Questionnaire-Priest version (OQ-P) included the following sections on priest’s views on: (a) causes of MDs. Twenty *“yes/no”* items grouped into five factors: biological; substance-abuse; stress-related; traumatic; and, supernatural causes of MD; (b) recommended professionals. Six *“yes/no”* items on health and religious professionals to be involved in the treatment of MD; (c) psychosocial consequences of MDs. Twenty-seven items, rated on a 3-point scale from 1= “*not true*” to 3= “*completely true*”, and grouped into the following thirteen factors: c.1) possibility of recovery in MDs (1 item); c.2-c.3) usefulness of pharmacological and psychological therapies in MDs (1 item, each); c.4) need for long-term pharmacological therapies (3 items); c.5-c.6) usefulness of prayer and exorcism as therapies for MDs (1 item, each); c.7) poor insight of people with MDs into their condition (2 items); c.8-c.9) perception of social distance from and dangerousness of people with MDs (5 items and 2 items, respectively); c.10) difficulties of people with MDs in affective relationships (2 items); c.11) easy recognizability of people with MDs (1 item); c.12) participation of people with MDs in parish activities and sacraments (4 items); c.13) discriminatory behaviors in religious celebrations (3 items). Regarding the factor named “recognizability” (c.11), it refers to respondent’s views about people with MDs as easy to recognize on the basis of appearances, gestures and behaviors considered unusual and/or peculiar to a given “type” of people. Concerning the factor named “discriminatory behaviors in religious celebrations” (c.13), it refers to respondent’s views about the opportunity to treat people with MD differently from other believers during the celebrations (i.e., separating them, supervising them, or perceiving them as a source of discomfort for other believers). In this factor’s items the term “discriminatory” is not used to avoid potential bias in the answers. The psychometric properties of the OQ-P, previously tested on the global sample of 559 priests, were found to be satisfactory (QO-P section a: Confirmatory Factor Analysis (CFA), χ^2^(558) = 341.86, *df* 160, *p* < .001; non-normed fit index [NNFI] = 0.93; comparative fit index [CFI] = 0.94; root mean square error of approximation [RMSEA] = 0.04, 90% C.I. (0.04; 0.05); standardized root mean square residual [SRMR] = 0.05; all factor loadings significant at *p* < .001 level; Cronbach’s *α* values of the factors ranging from 0.48 to 0.75. Section c: CFA, χ^2^(559) = 559.13, *df* 252 *p* < .05; non-normed fit index [NNFI] = 0.91; comparative fit index [CFI] = 0.93; root mean square error of approximation [RMSEA] = 0.05, 90% C.I. (0.04; 0.05); standardized root mean square residual [SRMR] = 0.05; all factor loadings significant at p < .001 level. Cronbach’s *α* values ranging from 0.52 to 0.84). For each OP-Q factor, an average score is computed. Single factor scores are not combined into a total overall score as not all factors correlated significantly with each other (Magliano et al., 2021). The study investigated the relationships between priests’ prejudices about people with schizophrenia and depression and the priests’ own opinions about whether people with MD should participate in religious activities and be treated like other churchgoers during celebrations. Therefore, OQ-P factors referring to priests’ prejudices toward people with MDs as individuals were included, while OP-Q factors referring to people with MDs as patients, e.g., those exploring views of biopsychosocial treatments, care from a religious perspective, and prognosis were excluded. Only the following six OQ-P factors (including a total of 17 items) were analyzed: poor insight of people with MDs into their condition (mean ± sd, schizophrenia group: 2.23 ± 0.50; depression group: 2.18 ± 0.50); perception of social distance from people with MDs (2.24 ± 0.41; 2.19 ± 0.49); perception of dangerousness (1.98 ± 0.49; 1.97 ± 0.47); easy recognizability of people with MDs (2.00 ± 0.68; 1.93 ± 0.70); participation of people with MDs in parish activities and sacraments (2.43 ± 0.37; 2.50 ± 0.38); discriminatory behaviors in religious celebrations (1.45 ± 0.48; 1.38 ± 0.46; mean scores reported in Magliano et al., 2021). Item-level information (for17 items) and factor-level data (for six factors) were included in analyses.

### Statistical Analyses

Frequencies and percentages were computed on each of the 17 items included in the following six OQ-P factors: poor insight of people with MDs into their condition; perception of social distance from people with MDs; perception of dangerousness; easy recognizability of people with MDs; participation of people with MDs in parish activities and sacraments; discriminatory behaviors in religious celebrations. As preliminary step of the multiple-group structural equation model, in each diagnostic group (schizophrenia and depression), Pearson’s correlation coefficient was computed to explore bivariate relations between the mean scores of the six OQ-P factors mentioned above and the community size of the municipalities where the churches were located (≤ 5.000 inhabitants; 5.001-10.000; 10.001-50.000; 50.001-100.000; >100.000 inhabitants). A multiple-group structural equation model was used to test the hypothesized relations among the following OQ-P factors: perception of social distance from people with MDs; perception of dangerousness; easy recognizability of people with MDs (independent observed variables) with the following OQ-P factors: participation of people with MDs in parish activities and sacraments; discriminatory behaviors in religious celebrations (dependent observed variables). In the model, type of disorder (schizophrenia and depression) was used as grouping variable (moderator) and community size was included as covariate influencing all the observed variables. Model fit was determined by computing the following indexes: Comparative Fit Index (CFI; Bentler, 1990), Tucker–Lewis index (TLI; Tucker & Lewis, 1973), and Root Mean Square Error of Approximation (RMSEA; Browne & Cudeck, 1993). A CFI and TLI ≥ 0.90, and RMSEA ≤ 0.08 indicate a model’s acceptable fit to the data (Hu & Bentler, 1999). To test the equivalence of the structural parameters across the two diagnostic groups (schizophrenia and depression), as first step of the analysis, parameters were freely estimated. As second-step of the analysis, structural paths and correlations were constrained to be equal across groups. The Satorra–Bentler chi-square difference test (ΔSBχ^2^) was used to test the relative fit of nested models (Satorra, 2000). A non-significant ΔSBχ^2^ led to choose the model with constrained structural paths and correlations (i.e., the moderator does not influence the relationships among the variables). Bivariate correlations were performed by SPSS version 21 (IBM, 2012). Multiple-group structural equation models were computed by Mplus 3, using the Maximum Likelihood Estimation (MLE; Lee & Seo, 2018). Statistical significance was set at p < .05.

## Results

### Frequencies of the Answers to the OQ-P Items in the Schizophrenia Group and the Depression Group

As shown in Table 1, in the schizophrenia group and the depression group, most of the priests believed that it was *‘completely true’* or *‘partially true’* that individuals with a condition like that reported in the clinical description were *“easy to recognize****”*** (schizophrenia group: 75.9%; depression groups: 69.9%), *“kept at a distance by the others”’*, (84.2%; 76.0%), and *‘dangerous to others’* (77.0% and 72.0%). Most priests thought it was *‘completely true’* or *‘partially true’* that people with these disorders were “*reliable when they confess”* (88.1% and 90.5%) and able to be “*witnesses in the sacraments (e.g., marriage)”* (87.4% in both groups). 51.8% of priests in the schizophrenia group and 43.9% of priests in the depression group believed that, during religious celebrations, people with MDs created *“discomfort to other churchgoers”*, and 46.6% and 37.1% of priests believed that these individuals “*should be supervised”*.

**Table 1:** Priests’ views of people with schizophrenia (N = 282) and depression (N = 277): frequencies of the 17 items included in the six OQ-P factors analyzed

Items	Schizophrenia Group (N = 282)						Depression group (N = 277)					
	Not true		Partially true		Completely true		Not true		Partially true		Completely true	
	N	%	N	%	N	%	N	%	N	%	N	%
§ are easy to recognize	64	24.1	136	51.1	66	24.8	78	30.1	121	46.7	60	23.2
§ are unpredictable	33	12.9	147	57.4	76	29.7	41	16.1	134	52.8	79	31.1
§ are kept at distance by the others	43	15.8	157	57.5	73	26.7	65	24.0	118	43.5	88	32.5
People does not know how to behave with §	21	7.6	125	45.5	129	46.9	25	9.4	128	47.9	114	42.7
People does not understand the difficulties experienced by §	23	8.4	137	49.8	115	41.8	24	9.0	125	46.8	118	44.2
People is frightened by §	29	10.9	148	55.4	90	33.7	68	25.8	118	44.7	78	29.5
§ are dangerous to themselves	34	13.5	170	67.5	48	19.0	29	11.3	173	67.6	54	21.1
§ are dangerous to others	58	23.0	161	63.9	33	13.1	71	28.1	151	59.7	31	12.3
§ do not realize that they are ill	20	7.7	154	59.2	86	33.1	37	14.1	154	58.6	72	27.4
§ do not realize when they become unwell	34	13.2	131	51.0	92	35.8	33	12.9	132	51.6	91	35.5
§ who are now well, may be responsible for parish activities (e.g. catechesis, etc.).	25	9.6	113	43.3	123	47.1	21	8.0	91	34.7	150	57.3
§ are reliable when they confess	29	11.9	150	61.5	65	26.6	23	9.5	140	57.9	9	32.6
§ can be witnesses in the sacraments (e.g. marriage)	33	12.7	94	36.2	133	51.2	33	12.7	68	26.2	159	61.2
§ can receive the Eucharist	7	2.5	41	14.7	231	82.8	10	3.6	37	13.5	228	82.9
During religious celebrations, §create discomfort to other churchgoers	128	48.1	119	44.7	19	7.1	150	56.2	100	37.5	17	6.4
During religious celebrations, §should be separated from other churchgoers	228	82.6	36	13.0	2	4.3	229	83.9	32	11.7	12	4.4
During religious celebrations, §should be supervised	148	53.4	109	39.4	20	7.2	170	63.0	85	31.5	15	5.6

§Individuals with a condition like that reported in the clinical description

### Bivariate Correlations Among the OQ-P Factors in the Schizophrenia Group and the Depression Group

In both the schizophrenia group and the depression group, priests’ perception of dangerousness and of social distance from people with MDs, and community size positively correlated with priests’ views regarding the opportunity to treat persons with MD differently from other churchgoers during religious celebrations (OQ-P factor “Discriminatory behaviors in religious celebrations”; Table 2). In the schizophrenia group, priests’ views that people with this disorder are easy to recognize negatively correlated with priests’ views about the opportunity that people with this disorder would participate in parish activities and sacraments. Furthermore, priests’ perception of dangerousness, easy recognizability and poor insight of people with schizophrenia positively correlated with priests’ perception of social distance from people with this disorder by the others. Moreover, in the schizophrenia group, priests’ views of people with this disorder as having poor insight negatively corelated with priests’ views regarding the participation of these people in parish activities and sacraments. In the depression group, priests’ perception of dangerousness of people with the disorder and community size negatively correlated with priests’ views regarding the participation of people with depression in parish activities and sacraments. Moreover, priests’ conviction that people with depression have poor insight and are easy to recognize positively correlated with priests’ views about the opportunity to treat people with this disorder differently from other churchgoers in religious celebrations. Finally, priests’ perception of people with depression as dangerous positively correlated with priests’ views of people with this disorder as easy to recognize and with population size.

**Table 2:** Correlations among the OQ-P factors and the community size in the schizophrenia (N = 282) and the depression group (N = 277)

OQ-P factors	1	2	3	4	5	6	7
1. Community size	–	.12^a^	− .05	.02	.01	− .15^b^	.29^e^
2. Perception of dangerousness	.06	–	.33^e^	.08	.21^e^	− .24^e^	.26^e^
3. Perception of social distance	− .02	.30^e^	–	.20^d^	.33^e^	− .11	.14^a^
4. Poor insight	− .07	.07	.18^c^	–	.01	− .07	.12^a^
5. Easy recognizability	− .04	.02	.20^d^	.02	–	− .15^b^	.15^b^
6. Participation in parish activities and sacraments	− .07	− .09	− .07	− .14^a^	− .25^e^	–	− .29^e^
7. Discriminatory behaviors in religious celebrations	.26^e^	.27^e^	.15^b^	.09	.09	− .22^e^	–

Correlations for schizophrenia are below the diagonal, correlations for depression are above the diagonal

^a^p < .05; ^b^p < .01; ^c^p < .005; ^d^p < .001; ^e^p < .0001

### Multiple-Group Structural Equation Model

**Fig. 1:**
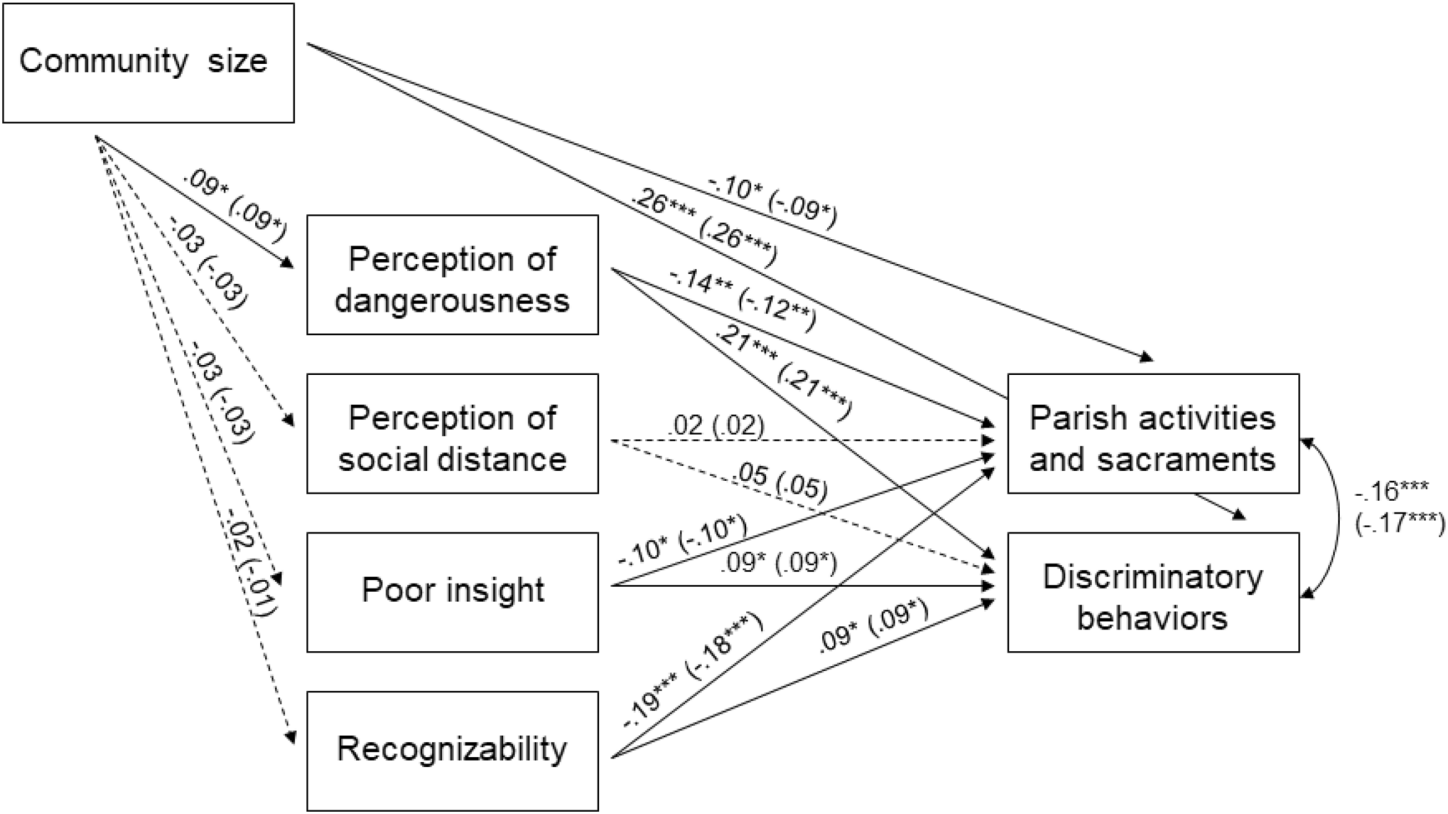
Multiple-group (schizophrenia and depression) structural equation model. Relations between priests’ perception of dangerousness, social distance, poor insight, easy recognizability of people with MDs and priests’ views regarding the participation of people with MDs in parish activities and sacraments, and the opportunity to adopt discriminatory behaviors toward this group of people during religious celebrations. Standardized path coefficients of the constrained model (Δχ^2^(21) = 18.20, p > .05). Parameters for schizophrenia are shown without brackets, parameters for depression are shown in brackets. Significant correlations between dangerousness with perception of social distance (schizophrenia group: β = 0.33, p < .001; depression group: β = 0.31, p < .001) and recognizability (β = 0.12, p < .01 ; β = 0.13, p < .01); between perception of social distance with insight (β = 0.20, p < .001; β = 0.18, p < .001); and recognizability (β = 0.28, p < .001; β = 0.25, p < .001); non-significant correlations between insight with perception of dangerousness (β = 0.07, p > .05 ; β = 0.08, p > .05); and recognizability (β = 0.01, p > .05. ; β = 0.01, p > .05). *p < .05 **p < .01 ***p < .001

Using the multi-group structural equation model, at the first step of the analysis the relationships between the observed variables were freely estimated simultaneously in the schizophrenia and the depression group. The fit indices of the unconstrained model were as follows: χ^2^(0) = 0, *p* = .0, RMSEA = 0.0 (0.0; 0.0), TLI = 1, CFI = 1 (saturated model). During the second step of the analysis, structural paths and correlations were constrained to be equal across the diagnostic groups. The fit indices for the constrained model were as follows: χ^2^(21) = 18.20, *p* = .63, RMSEA = 0.0 (0.0; 0.04), TLI = 1, CFI = 1. The delta chi-square statistic showed that the fit of the constrained model across diagnostic groups was significantly better than the fit of the unconstrained model, Δχ^2^(21) = 18.20, *p* > .05. Therefore, the constrained model with total invariance across diagnostic groups was adopted. In line with our hypothesis (Fig. 1), priests’ perceptions of people with MDs as dangerous, having poor insight and easy to recognize were negatively correlated with priests’ acceptance of churchgoers with MDs in parish activities and sacraments. The same predictors were positively correlated with priests’ beliefs about the opportunity to adopt discriminatory behaviors toward individuals with MDs in religious celebrations. Conversely, priests’ perception of social distance from people with MD was not significantly associated with priests’ beliefs about the opportunity that this group of people would participate in parish activities or sacraments nor that persons with MD should be treated differently from other churchgoers during religious celebrations. All correlations among priests’ perception of people with MDs as dangerous, kept at social distance, and as having poor insight and easy to recognize were positive and statistically significant (p < .05), except the correlations of priests’ conviction that persons with MD have poor insight with priests’ perception of such persons as dangerous and easy to recognize (p > .05). Priests’ conviction that persons with MD should be treated differently from other churchgoers during religious celebrations were significantly and negatively correlated with priests’ views regarding the opportunity that people with MDs would participate in parish activities and sacraments. Community size, included in the model as a covariate influencing all variables, was positively associated with priests’ perception of persons with MD as dangerous and to be treated differently from the other churchgoers during religious celebrations. Moreover, community size was negatively associated with priests’ views about the opportunity for persons with MD to participate in parish activities and sacraments. Overall, the model explained 8% of variance in the schizophrenia group and 7% of variance in the depression group for priests’ views regarding the participation of people with MDs in parish activities and sacraments and 15% of variance in both the groups for priests’ beliefs about the opportunity to adopt discriminatory behaviors toward persons with MD during religious celebrations.

## Discussion

### Interpretation of the Results

This study showed that priests’ attitudes regarding people with MDs participating in religious activities are significantly related to priests’ views about this group of people as dangerous, easy to recognize and as having poor insight. The study also revealed that community size has both a direct effect on priests’ attitudes and an indirect effect through priests’ perception of dangerousness in people with MDs. Finally, the study revealed that the above-mentioned relationships do not differ by the type of disorder. This is only true when these relationships are analyzed within a multiple-group structural equation model (as shown by the non-significance of the delta χ^2^). It may seem odd that the relationships between the variables were equivalent between the two groups when analyzed in the path analysis, whereas they were different when analyzed with the bivariate correlations in each group. This apparent discrepancy can be explained by the fact that the correlations analyze two variables at a time and in individual samples, whereas the multigroup model analyses the relationships simultaneously between all variables within and between groups. Interestingly, bivariate correlations showed a relationship between perceived dangerousness and population size only in the depression group. One possible explanation could be that in rural centers, close relationships among community members would facilitate contact with people with depression, a mental disorder with high social acceptance, mitigating perceived dangerousness.

In highly populated areas, priests were more convinced of the dangerousness of individuals with MDs, more reluctant to acknowledge the capacities of these people to participate in religious activities, and more inclined to treat them differently from the other churchgoers during celebrations. In highly-populated areas, priests likely have less direct contact with believers (Ellison et al., 2006). This, in its turn, may increase priests’ desire for social distance from people with MDs (Lee & Seo, 2018). Moreover, the relationships of community size with priests’ perceived dangerousness in people with MDs and priests’ acceptance of these people in religious contexts could be explained by the higher public perception of social insecurity in large urban areas vs. small towns and rural areas. In highly-populated areas people are more uncertain that “others” would intervene to help them if needed (Triventi, 2008). Perception of insecurity can fuel individuals’ fears of being exposed to socially dangerous situations, including those stereotypically associated with MDs. Conversely, in small towns and villages, close social relationships between residents can foster social support and control of disturbing behaviors. Such conditions could facilitate both the acceptance of people with MDs in the community and their participation in religious practices.

In addition to perception of dangerousness, priests’ convictions that people with MDs are easy to recognize and unaware of their condition influence priests’ attitudes about these individuals’ participation in religious activities (e.g., being trustworthy in confession and witness in sacraments) and priests’ views about the opportunity to adopt discriminatory behaviors toward persons with MD during celebrations. These data are in line with those reported by Leavey et al. (2007) about the tendency to exclude individuals with MDs from religious practices “for the well-being of the community”. A notable percentage of priests believed that people with MDs would create discomfort for other churchgoers and that they need to be supervised. These attitudes – if expressed by the priests in the parish context - may facilitate avoidance of people with MDs by the other churchgoers and the self-exclusion of people with these disorders from religious contexts (Thornicroft et al., 2009). This is particularly worrying in Southern Italy, where church attendance is considered a well-established cultural trait that transcends religious motivations and takes on an important social meaning (Cartocci, 2011). The hypothesis regarding the relationship between need for social distance from people with MDs and priests’ attitudes about the participation of such people in religious practices was not confirmed. This result may be since this factor measures social distance attributed by the priest to ordinary people and does not reflect the priest’s own desire for social distance. The relationships between priests’ prejudices about people with MDs and priests’ attitudes about the participation of this group of people in religious activities was in part in line with the results of a previous study on GPs’ views of individuals with schizophrenia (Magliano et al., 2017). In that study, GPs that were surer that these people were dangerous more firmly believed that they should be discriminated against in non-psychiatric hospital wards and were incapable of reporting their health problems to doctors.

The results of this study may contribute to a greater understanding of the relationships among the components of stigma, particularly those between priests’ views of people with MD and priests’ attitudes toward the participation of this group of people in religious activities. These results may have practical relevance and point to potential areas for intervention within religious communities. Countering priests’ stereotypes about dangerousness and poor insight to MDs could reduce the proportion of priests who tend to discriminate against churchgoers because of their MDs (Thornicroft et al., 2016). Providing priests with balanced information on the biopsychosocial etiology of MDs, successful treatments, and positive prognosis, can help priests to perceive individuals with MDs, particularly those with schizophrenia, as worthy persons rather than a threat for other churchgoers. In its turn, educating priests can facilitate the acceptance of believers with DM by other churchgoers by providing them with a supportive social network (Oman & Thoresen 2005). Involving individuals recovered from a MD as responsible for parish activities may act as a facilitator in recovery process (Mohr et al. 2006) and reduce the perception of people with MDs as dangerous among other churchgoers. In people with MD, participation in parish activities can also facilitate self-esteem and empowerment (in Webb et al. 2011). This is particularly relevant in Italy, a predominantly Catholic country where the network of dioceses and parishes is very extensive, and religious volunteering is widespread nationwide (Cartocci, 2011). These results can be of interest for public health and clinical practitioners. Educating priests about MDs can foster collaboration between clergy and mental health professionals. This collaboration could be promoted through outreach initiatives aimed at the entire parish community, such as informative meetings on MDs held by mental health professionals in parishes (Stetz et al., 2011). Ensuring participation in religious practices by believers with MDs is perhaps even more important in this period where the COVID-19 pandemic has led to the reduction of many social activities while churches have remained open even in lockdown.

### Strengths and Limitations of the Study

The novelty of the present study is that, using a multifactorial model, it has examined the relationships between priests’ views of people with MDs as dangerous, kept at distance by the others, easy to recognize, and unaware of their condition and priests’ attitudes towards churchgoers with these disorders. The above-mentioned relationships have been investigated simultaneously testing the moderator role of diagnostic group and analyzing whether the relationships varied according to community size. This methodologically new approach based on path analysis had two main advantages over conducting separate analyses for schizophrenia and depression groups. First, it provided a test for significance of any difference found between the groups. Second, since no difference was detected between the schizophrenia group and the depression group, the simultaneous analysis provided more accurate parameter estimates than would be obtained from two single-group analyses. Further strengths are the large sample size, the face-to-face data collection, and the low refusal rate (8%). The use of a validated self-reported questionnaire is a further strength, also facilitating replication of the survey. The study has a few limitations, suggesting caution in interpretation of its results. In particular, it should be considered that: (a) the inclusion of priests from only Southern Italy may limit the generalizability of the results to other geographical contexts. In this geographical area, the percentages of Catholics and of Catholics regularly attending churches are higher than in the country as a whole (78.5% vs. vs. 66.7%; 30.9% and 25.1%; Doxa, 2019; ISTAT, 2012); (b) the cross-sectional design of the study does not permit inferences regarding the effects of examined variables; c) the results refers to priests’ views and are not based on actual observations of their behaviors; therefore, they may not reflect priests’ real acceptance of churchgoers with MDs; d) the fact that, although this study allows for generalizability of the results to different population contexts (from metropolitan areas to small villages), it only considered Catholic clergy. Future studies are needed to clarify whether these findings are common to different religious contexts. Some of these limitations will be addressed in further studies at their planning stage.

## Data Availability

The data that support the findings of this study are available from the corresponding author upon reasonable request, which must include a protocol and statistical analysis plan and not be in conflict with our publication plan.

## Appendix 1

Some people sometimes seem unable to distinguish between things that really happen and are experienced by other people, and things that happen only in their mind. Sometimes, these people believe or say things that seem bizarre or absurd to other people, or hear voices, smell things, or see images that other people do not. Sometimes, these people may have difficulty expressing their feelings or behaving appropriately (for instance, they may cry in response to a positive event, or may appear happy following an unpleasant one), or they may remain shut up in their house for a long time or talk very little or not at all. They behave as if they lived in a world of their own, apparently without interest in anything or anybody. Sometimes they may have muddled thoughts, may invent odd or incomprehensible words, may lose the thread of the speech, or they may jump from one issue to another with no apparent reason.

## Appendix 2

Some people sometimes feel sad, down, unable to feel pleasure, or to have interest for those activities they liked in the past. Sometimes, these people feel incompetent, may believe to be derided by the others, and make themselves feel guilty for trivial things. These people may have no hope for future and when their feelings of sadness and worthlessness become unbearable, they may decide to stop living. Sometimes, these people may have difficulties in eating and sleeping regularly and may feel poor concentrated or physically tired. Other times, they may feel irritable and get annoyed with the others for unimportant things.

## References

[CR1] Anshel MH, Smith M (2014). The role of religious leaders in promoting healthy habits in religious institutions. Journal of Religious and Health.

[CR2] Aramouny C, Kerbage H, Richa N, Rouhana P (2020). Knowledge, attitudes, and beliefs of catholic clerics’ regarding mental health in Lebanon. Journal of Religious and Health.

[CR3] Bentler PM (1990). Comparative fit indexes in structural models. Psychological Bulletin.

[CR4] Bonelli RM, Koenig HG (2013). Mental Disorders, Religion and Spirituality 1990 to 2010: A systematic evidence-based review. Journal of Religious and Health.

[CR5] Browne MW, Cudeck R, Bollen KA, Long JS (1993). Alternative ways of assessing model fit. *Testing structural equation models*.

[CR6] Cartocci R (2011). Geografia dell’Italia cattolica (Geography of Catholic Italy).

[CR41] DOXA. (2019). *Religiosità e ateismo. Indagine demoscopica sulla popolazione italiana (Religiosity and atheism. Demoscopic survey of the Italian population).* Retrieved from https://www.uaar.it/doxa2019/

[CR7] Ellison CG, Vaaler ML, Flannelly KJ, Weaver AJ (2006). The clergy as a source of mental health assistance: What Americans believe. Review of Religious Research.

[CR8] Fallot RD (2007). Spirituality and religion in recovery: Some current issues. Psychiatric Rehabilitation Journal.

[CR9] Griffith JL, Myers N, Compton MT (2016). How can community religious groups aid recovery for individuals with psychotic illnesses?. Community Mental Health Journal.

[CR10] Hackett, C. (2011). *The Global Religious Landscape.* Pew Research Center. Retrieved from https://www.pewforum.org/2017/04/05/the-changing-global-religious-landscape/

[CR11] Hook JN, Worthington EL, Davis DE, Jennings DJ, Gartner AL, Hook JP (2010). Empirically supported religious and spiritual therapies. Journal of Clinical Psychology.

[CR12] Hu, J. T., & Bentler, P. M. (1999). Cut-off criteria for fit indexes in covariance structure analysis: Conventional criteria versus new alternatives. *Structural Equation Modeling, 6*(1), 1– 55. 10.1080/10705519909540118

[CR42] IBM Corp. Released (2012). *IBM SPSS Statistics for Windows*, Version 21.0. Armonk, NY: IBM Corp

[CR13] Igbinomwanhia NG, James BO, Omoaregba JO (2013). The attitudes of clergy in Benin City, Nigeria towards persons with mental illness. African Journal of Psychiatry.

[CR43] Istituto Nazionale Statistica ISTAT. (2012). *Pratica religiosa (Religious practice).* Retrieved from http://dati.istat.it/index.aspx?queryid=24349

[CR14] Istituto Nazionale Statistica, I. S. T. A. T. (2017). *Forme, livelli e dinamiche dell’urbanizzazione in Italia (Patterns, levels and dynamics of urbanisation in Italy).* Retrieved from https://www.istat.it/it/archivio/199520

[CR15] Lafuze JE, Perkins DV, Avirappattu GA (2002). Pastors’ perceptions of mental disorders. Psychiatric Services.

[CR16] Leavey G, Loewenthal K, King M (2007). Challenges to sanctuary: The clergy as a resource for mental health care in the community. Social Sciences and Medicine.

[CR17] Lee M, Seo M (2018). Effect of direct and indirect contact with mental illness on dangerousness and social distance. International Journal of Social Psychiatry.

[CR18] Link BG, Phelan JC, Bresnahan M, Stueve A, Pescosolido BA (1999). Public conceptions of mental illness: Labels, causes, dangerousness, and social distance. American Journal of Public Health.

[CR19] Magliano L, Citarelli G, Affuso G (2021). Views of catholic priests regarding causes, treatments and psychosocial consequences of schizophrenia and depression: A comparative study in Italy. Journal of Religious and Health.

[CR20] Magliano L, Punzo R, Strino A, Acone R, Affuso G, Read J (2017). General practitioners’ beliefs about people with schizophrenia and whether they should be subject to discriminatory treatment when in medical hospital: The mediating role of dangerousness perception. American Journal of Orthopsychiatry.

[CR21] Mohr S, Borras L, Nolan J, Gillieron C, Brandt PY, Eytan A, Huguelet P (2012). Spirituality and religion in outpatients with schizophrenia: A multi-site comparative study of Switzerland, Canada, and the United States. International Journal of Psychiatry and Medicine.

[CR22] Mohr S, Brandt PY, Borras L, Gilliéron C, Huguelet P (2006). Toward an integration of spirituality and religiousness into the psychosocial dimension of schizophrenia. American Journal of Psychiatry.

[CR23] Muthén LK, Muthén BO (2017). Mplus User’s Guide (version 8).

[CR24] Nolan JA, McEvoy JP, Koenig HG, Hooten EG, Whetten K, Pieper CF (2012). Religious coping and quality of life among individuals living with schizophrenia. Psychiatric Services.

[CR25] Oman D, Thoresen CE, Paloutzian RF, Park CL (2005). Do religion and spirituality influence health?. Handbook of the psychology of religion and spirituality.

[CR26] Pescosolido BA (2013). The public stigma of mental illness: what do we think; what do we know; what can we prove?. Journal of Health and Social Behavior.

[CR27] Russinova Z, Blanch A (2007). Supported spirituality: a new frontier in the recovery-oriented mental health system. Psychiatric Rehabilitation Journal.

[CR28] Satorra, A. (2000). Scaled and adjusted restricted tests in multi-sample analysis of moment structures. In: *Innovations in multivariate statistical analysis* (pp. 233–247). Boston, MA: Springer. 10.1007/978-1-4615-4603-0_17

[CR29] Shah R, Kulhara P, Grover S, Kumar S, Malhotra R, Tyagi S (2011). Relationship between spirituality/religiousness and coping in patients with residual schizophrenia. Quality of Life Research.

[CR30] Smolak A, Gearing RE, Alonzo D, Baldwin S, Harmon S, McHugh K (2013). Social support and religion: Mental health service use and treatment of schizophrenia. Community Mental Health Journal.

[CR31] Stetz K, Webb M, Holder M, Zucker A (2011). Mental health ministry: crating healing communities for sojourners. Journal of Religion, Disability & Health.

[CR32] Stull LG, Harness J, Miller M, Taylor A (2020). Attitudes about mental illness among seminary students: A qualitative analysis. Journal of Religious and Health.

[CR33] Thornicroft G, Brohan E, Rose D, Sartorius N, Leese M, INDIGO Study Group (2009). Global pattern of experienced and anticipated discrimination against people with schizophrenia: A cross-sectional survey. Lancet.

[CR34] Thornicroft G, Mehta N, Clement S, Evans-Lacko S, Doherty M, Rose D, Henderson C (2016). Evidence for effective interventions to reduce mental-health-related stigma and discrimination. Lancet.

[CR35] Triventi M (2008). . Segni di inciviltà sul territorio e paura del crimine. Un’analisi dei dati dell’Indagine sulla sicurezza dei cittadini (Signs of incivilities and fear of crime. An analysis of data from the Italian survey on citizens’ safety). Quaderni di Sociologia.

[CR36] Tucker LR, Lewis C (1973). A reliability coefficient for maximum likelihood factor analysis. Psychometrika.

[CR37] Webb M, Charbonneau AM, McCann RA, Gayle KR (2011). Struggling and enduring with God, religious support, and recovery from severe mental illness. Journal of Clinical Psychology.

[CR38] Weber SR, Pargament KJ (2014). The role of religion and spirituality in mental health. Current Opinions in Psychiatry.

[CR44] WHO - The world health report. (2001). *Mental health: New understanding. New Hope.* Retrieved from https://www.who.int/whr/2001/en/

[CR39] Wong-McDonald A (2007). Spirituality and psychosocial rehabilitation: empowering persons with serious psychiatric disabilities at an inner-city community program. Psychiatric Rehabilitation Journal.

[CR40] Yangarber-Hicks N (2004). Religious coping styles and recovery from serious mental illness. Journal of Psychology and Theology.

